# *TNFRSF12A* expression in stomach adenocarcinoma and its preliminary role in predicting immunotherapy response

**DOI:** 10.3389/fimmu.2025.1578068

**Published:** 2025-05-05

**Authors:** Lin-De Sun, Lin-Lin Zhang, Zheng Wan, Xiao-Dong Yang, Jing Yao, Ze-Long Yang, Lin Liu, Jun-Yan Liu

**Affiliations:** Department of General Surgery, The First Medical Center, Chinese People’s Liberation Army (PLA) General Hospital, Beijing, China

**Keywords:** *TNFRSF12A*, single-cell RNA sequencing, stomach adenocarcinoma, tumor microenvironment, immunotherapy

## Abstract

**Background:**

*TNFRSF12A* is abnormally expressed in various malignancies, especially in stomach adenocarcinoma (STAD), which is related to tumor invasiveness and prognosis of patients. This study examined the expression pattern of *TNFRSF12A* in STAD and predicted immunotherapy response.

**Methods:**

Data were derived from The Cancer Gene Atlas (TCGA), Gene Expression Omnibus (GEO), and Gene Expression Profiling Interactive Analysis (GEPIA) to analyze the expression pattern of *TNFRSF12A* in pan-cancer and STAD, as well as its correlation with clinical features. Biological pathways involved in *TNFRSF12A* were analyzed by “clusterProfiler” package. Immune cell infiltration was evaluated by “GSVA” and “CIBERSORT” packages. Immunotherapy response was assessed by TIDE score and tumor mutation burden (TMB) level. Expression level of *TNFRSF12A* in the single cell of STAD was analyzed by scRNA-seq. Finally, *in vitro* test detected the mRNA expression of *TNFRSF12A* in STAD cells, Wound healing and Transwell assays were performed to measure the capabilities of STAD cell to migrate and invade.

**Results:**

*TNFRSF12A* was highly expressed in STAD. However, *TNFRSF12A* expression did not shown significant difference in relation to clinical features. *TNFRSF12A* exhibited notably positive correlation with many carcinogenic signaling pathways and immune cells infiltration such as T cells and macrophages. High *TNFRSF12A* expression group showed a higher TIDE score, Exclusion score, and TMB level than the low *TNFRSF12A* expression group, which indicated that STAD patients with high *TNFRSF12A* expression responded more poorly to immunotherapy. *TNFRSF12A* showed a positive relation with most of immune checkpoint genes. By scRNA-seq analysis, *TNFRSF12A* was chiefly expressed in Fibroblasts and Mast cells of STAD. Further, *in vitro* assays verified the high expression of *TNFRSF12A* in STAD cells, and the migration and invasion capabilities of STAD cells were notably suppressed by *TNFRSF12A* silencing (*p*<0.05).

**Conclusion:**

The present study not only reveals the potential of *TNFRSF12A* as a therapeutic target for STAD, but also explores its great potential in STAD immunotherapy. This finding opens up a new way of thinking for the personalized treatment of STAD.

## 1 Introduction

As a frequently detected cancer globally (1, 2), gastric cancer (GC) has complicated pathogenesis, comprising environmental factor, genetic predisposition, and chronic inflammation (3–5). GC exhibits strong invasion and metastasis characteristics, which leads to the high incidence and mortality rates (6). Stomach adenocarcinoma (STAD) is a prevailing type of GC, accounting for about 90%, and has different molecular subtypes and clinical behaviors (7). Most patients have already been at the middle or advanced stages when diagnosed due to the nontypical clinical symptoms of early STAD (8). At present, endoscopic surveillance is the standard screening method that has made a breakthrough in the detection and therapy of STAD, but its high price and invasiveness is merely limited to high-risk patients (9). Surgical resection, chemotherapy, radiotherapy, and immunotherapy are commonly applied for STAD management (10). Nonetheless, the long-term survival probability of STAD is still disillusionary owing to tumor recurrence and metastasis (11). Immune checkpoint inhibitors (ICIs) have also been manifested to be resultful in controlling the development of STAD, yet they only benefit a small number of patients (12). Thus, the accurate diagnosis and effective treatment of STAD remains a serious challenge facing modern medicine; it is necessary to understand the pathogenesis of STAD and search for reliable markers.

With the deepening research on tumor microenvironment (TME), tumor-associated inflammation has been shown to regulate STAD proliferation, migration, and immune escape through cytokines and chemokines, which in turn affects patient survival (13–15). *TNFRSF12A*, alternatively known as Fn14, belongs to tumor necrosis factor (TNF) receptor superfamily and exerts its biological functions mainly by binding to its ligand TWEAK (16). *TNFRSF12A* can promote angiogenesis and regulate apoptosis, as well as also affect the immune escape ability of tumors by regulating immune cell infiltration in TME (17). It has been suggested that *TNFRSF12A* expression is abnormally upregulated in numerous carcinomas, such as colorectal cancer (18), glioma (19), and breast cancer (20), which is closely associated with tumor aggressiveness and patient prognosis. Particularly, *TNFRSF12A* has also been reported to be markedly overexpressed in GC tissues and cells, indicating a worse prognostic outcomes of GC (21). Given that STAD is a common subtype of gastric cancer, it is highly likely that TNFRSF12A also plays a critical role in the pathogenesis of STAD. Therefore, investigating the roles of *TNFRSF12A* in STAD is not only helpful to understand the development mechanism of STAD, but also could offer a novel therapeutic target in clinical practice.

We first acquired sample data from the public database to reveal the expression pattern of *TNFRSF12A* in pan-cancer and assess its correlation with patient prognosis and clinical features in STAD. Further, we analyzed the biological pathways involved in *TNFRSF12A* and evaluated the immune cell infiltration in STAD. TIDE score and TMB level were calculated to predict immunotherapy responses. Finally, the expression of *TNFRSF12A* in the single cell of STAD was assessed by scRNA-seq analysis. *In vitro* test was further conducted. This study confirmed the high expression of *TNFRSF12A* in STAD and the correlation of immune infiltration, suggesting that *TNFRSF12A* may be a therapeutic target for STAD. Meanwhile, the study also revealed the potential value of *TNFRSF12A* in the field of STAD immunotherapy, which provides key clues for in-depth investigation of the immunotherapeutic mechanism of STAD and the development of more effective therapeutic strategies.

## 2 Materials and methods

### 2.1 Data source and preprocessing

The RNA-seq data of *TNFRSF12A* in pan-cancer were obtained from the GEPIA database (http://gepia.cancer-pku.cn/).

Transcriptome data, clinical data, and mutation data of STAD were derived from the Cancer Gene Atlas (TCGA) database. Then, FPKM values were converted into TPM and log2 conversion was performed. The samples with complete survival data were reserved, including 32 normal samples and 350 tumor samples.

Clinical information and RNA-seq data of GSE66229 dataset as well as scRNA-seq data of GSE167297 dataset were all acquired from the GEO database. For GSE66229 dataset, the probes were transformed into gene symbols based on the annotation information, and the gene with the highest average expression was selected when corresponding to duplicate gene symbols or multiple probes.

### 2.2 Gene set enrichment analysis

Samples in TCGA-STAD cohort were divided by the median expression of *TNFRSF12A* into high and low expression groups to examine the relationship between *TNFRSF12A* expression and biological pathways. Kyoto Encyclopedia of Genes and Genomes (KEGG) enrichment analysis was conducted by GSEA using the gseKEGG function in the “clusterProfiler” R package (22), and the top5 KEGG pathways were screened based on the normalized enrichment score (NES). HALLMARK pathway scores were calculated using the “GSVA” R package (23), and the gene sets were derived from the MSigDB (https://www.gsea-msigdb.org/gsea/msigdb/). The relationship between HALLMARK pathways and *TNFRSF12A* expression was analyzed (*p*<0.05).

### 2.3 Immune cell infiltration analysis

Single sample GSEA (ssGSEA) was applied to calculate 28 types of tumor-infiltrating lymphocytes (TILs) scores with the “GSVA” R package (24), and the gene sets were obtained from a previous study (25). The “CIBERSORT” R package was employed to quantify the abundance of 22 types of immune cells in TCGA-STAD cohort (26). The correlation between immune cell infiltration and *TNFRSF12A* expression was analyzed.

### 2.4 Immunotherapy response assessment

The TIDE score and Exclusion score was calculated by TIDE algorithm (27), predicting the response of STAD patients to ICIs therapy. Moreover, the expressions of immune checkpoint genes and TMB were served as potential predictors of immunotherapy response. TMB was counted using the “maftools” R package (28).

### 2.5 Single cell data analysis

The scRNA-seq data of each sample in GSE167297 dataset was read by the Read10X function in the “Seurat” R package (29), retaining the cells with gene numbers of 200–2500 and mitochondrial gene ratio of <10%. Next, the SCTransform function was employed for normalization, and after principal component analysis, the “harmony” R package was applied for removing batch effects (30). Subsequently, tSNE dimensionality reduction was conducted by the RunTSNE function. The FindNeighbors and FindClusters functions (parameters: dims=1:20 and resolution=0.1) were employed to cluster the cells clustering. Cell types were annotated according to the marker genes offered by CellMarker2.0 database (31, 32).

### 2.6 Cell cultivation and transfection

Human gastric mucosa epithelial cell line GES-1 (CBP60512, Nanjing Cobioer Biotechnology Co., China) and STAD cell line AGS (CBP60476, Cobioer, China) were acquired beforehand. GES-1 cell line was cultivated in DMEM (CBP60512M, Cobioer, China) containing 10% fetal bovine serum (FBS), and AGS cell line was grown in RPMI-1640 (CBP60476M, Cobioer, China) encompassing 10% FBS. All cells were stored at an incubator of 5% CO_2_ and 37˚C. All cells were tested for mycoplasma contamination, identified as contamination-free and certified as short tandem repeat (STR).

Subsequently, to silence the *TNFRSF12A* in AGS cells, the small interfering (si) RNA of *TNFRSF12A* (si-*TNFRSF12A*#1 from Merck KGaA, Darmstadt, Germany and si-*TNFRSF12A#2*: 5’-AGGGAGAATTTATTAATAAAAGA-3’, Sangon Biotech (Shanghai) Co., Ltd., Shanghai, China) and negative control (si-NC) was applied to transfect the AGS cells through Lipofectamine 2000 (Invitrogen, USA).

### 2.7 Real-time quantitative PCR

Total RNA of GES-1 and AGS cells was collected utilizing the TRIzol reagent (15596026, Thermo Fisher). Then, the PrimeScript reverse transcriptase reagent Kit (RR037Q, Takara, Japan) was used to synthesize cDNA. RT−qPCR was carried out using the SYBR Green Universal Master Mix (4364344, Thermo Fisher) in a QuantStudio 3 Real-Time PCR System (Thermo Fisher). The primer pairs were designed by Sangon Biotech (Shanghai) Co., Ltd. The primer sequences for *TNFRSF12A* were 5’-GACCTGGACAAGTGCAT-3’ (forward) and 5’-GGTGGTGAACTTCTCTCTC-3’ (reverse), for *TPSAB1* were 5’-CACCCACAGTTCTACACC-3’ (forward) and 5’-GGATCCAGTCCAAGTAGTAG-3’ (reverse), for *DCN* were 5’-ATGAAGGCCACTATCATCCTCC-3’ (forward) and 5’-GTCGCGGTCATCAGGAACTT-3’ (reverse), for *GAPDH* were 5’-CTGGGCTACACTGAGCACC-3’ (forward) and 5’-AAGTGGTCGTTGAGGGCAATG-3’ (reverse). *GAPDH* was applied as the housekeeping gene, and the relative mRNA expression levels of *TNFRSF12A*, *TPSAB1*, and *DCN* were calculated by 2^-ΔΔCT^ method (33).

### 2.8 Wound healing assay

The ability of AGS cells to migrate could be measured by wound healing assay (34). In short, the transfected AGS cells were planted into 6-well plates and cultivated until a uniform monolayer was formed. An aseptic micropipette was applied to form scratch wound on the surface of AGS cells. After that, the wound images at 0 and 48h were obtained under an inverted microscope (Primo vert, ZEISS, Germany) and the wound closure rate of AGS cells was estimated employing the ImageJ software (version 1.42G) (32).

### 2.9 Transwell assay

The ability of AGS cells to invade was measured by Transwell assay (35). Briefly, diluted Matrigel (Corning, USA) was pre-coated in the Transwell chamber (8.0μm, Corning, USA). Next, the transfected AGS cells (2×10^4^ cells/well) were suspended in 200µL serum-deleted RPMI-1640 medium and cultured in the upper chamber, whereas the lower chamber was supplemented with 600µL RPMI-1640 medium encompassing 10% FBS. After 48h incubation, the invaded AGS cells were fixed in 4% paraformaldehyde (ZY640017RE, Zeye, Shanghai, China) for 10 minutes, stained by 0.1% crystal violet (ZY-9248, Zeye, Shanghai, China) for 5 min, and washed twice using phosphate buffer solution. Under the Primo vert inverted microscope (ZEISS, Germany), the number of invaded AGS cells was counted from six randomly picked fields.

### 2.10 Statistical analysis

R software (version 4.2.0) and GraphPad Prism (version 8.0) were applied for statistical analysis. All experiments were performed in triplicate and data were shown as mean ± standard deviation. The difference between two continuous variables was compared by t test. For experiments involving three or more variables, we employed the analysis of variance (ANOVA) test to assess the overall differences among the groups. Subsequently, a Sidak’s multiple comparisons test was conducted to determine the specific pairwise differences between each group. The correlation analysis was conducted by Spearman method. To evaluate the survival differences, Kaplan-Meier (K-M) survival analysis was performed by “survminer” R package (36). A *p*<0.05 denoted a statistical significance level.

## 3 Results

### 3.1 *TNFRSF12A* expression in pan-cancer and its correlation with clinical features in STAD

**Figure 1:**
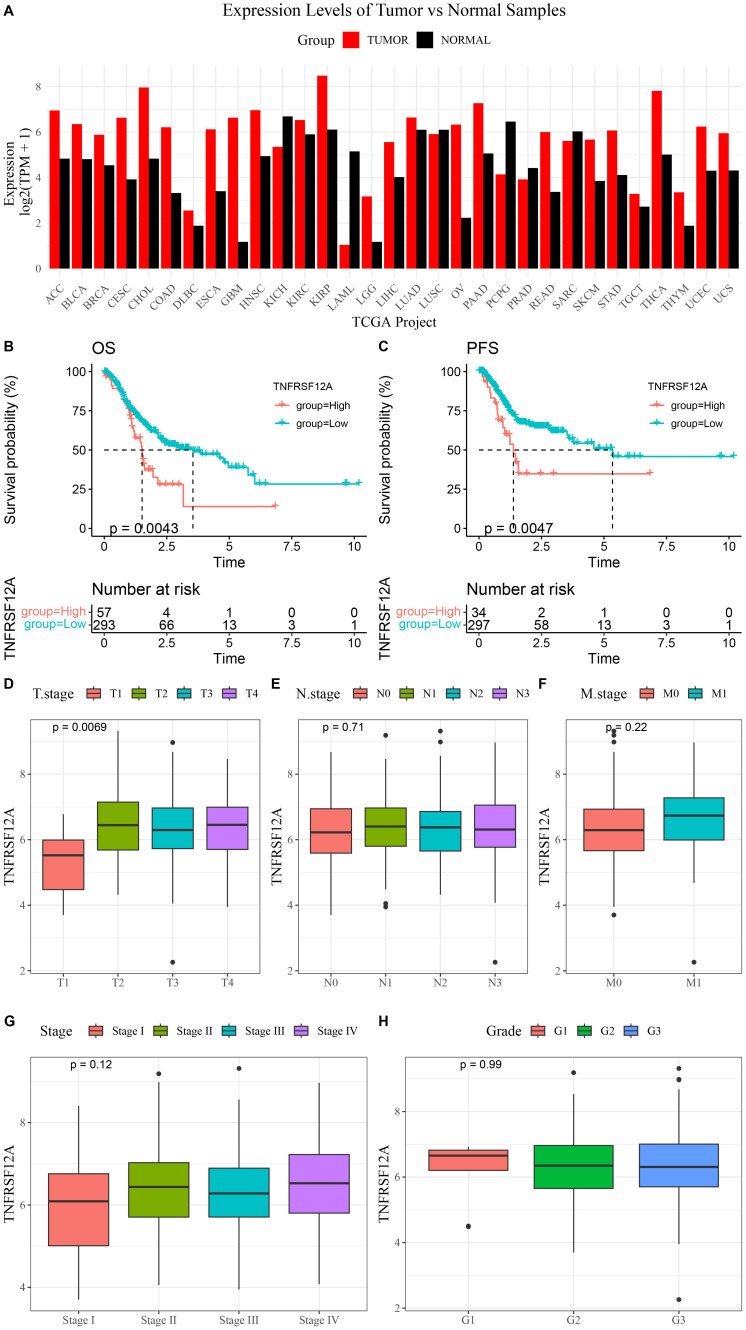
Expression of *TNFRSF12A* in pan-cancer and its correlation with clinical features in STAD. **(A)** Expression levels of *TNFRSF12A* in pan-cancer; **(B)** Kaplan-Meier (K-M) curve of overall survival (OS) for high and low *TNFRSF12A* expression groups in STAD; **(C)** K-M curve of progression-free survival (PFS) for high and low *TNFRSF12A* expression groups in STAD; **(D)** Relationship between *TNFRSF12A* expression and T stages in STAD; **(E)** Relationship between *TNFRSF12A* expression and N stages in STAD; **(F)** Relationship between *TNFRSF12A* expression and M stages in STAD; **(G)** Relationship between *TNFRSF12A* expression and Stage in STAD; **(H)** Relationship between *TNFRSF12A* expression and Grade in STAD.

The expression data of *TNFRSF12A* was acquired from the GEPIA database. It was found that compared to normal samples, *TNFRSF12A* was high-expressed in most tumors, such as STAD, colon adenocarcinoma (COAD), lung adenocarcinoma (LUAD), liver hepatocellular carcinoma (LIHC), and glioblastoma (GBM) (**Figure 1A**). Further, K-M curves demonstrated that the progression-free survival (PFS) and overall survival (OS) rates of high *TNFRSF12A* expression group in STAD were all lower than that of low *TNFRSF12A* expression group (**Figures 1B, C**). Meanwhile, the relationship between *TNFRSF12A* expression and clinicopathologic characteristics in STAD was analyzed by Spearman method. The *TNFRSF12A* expression was significantly different among different T stages (*p*=0.0069), and the expression of *TNFRSF12A* in T2, T3, and T4 stages was higher than that in T1 stage (**Figure 1D**). However, the expression of *TNFRSF12A* in different N stages (N0, N1, N2, N3), M stages (M0, M1), Stage (I, II, III, IV), or Grade (G1, G2, G3) showed no significant difference (*p*>0.05) (**Figures 1E–H**). This result implies that *TNFRSF12A* is highly expressed in most of the tumors, but there is no significant relationship between *TNFRSF12A* expression and clinicopathological features in STAD.

### 3.2 *TNFRSF12A* expression and its relationship with clinical features in GSE66229 dataset

**Figure 2:**
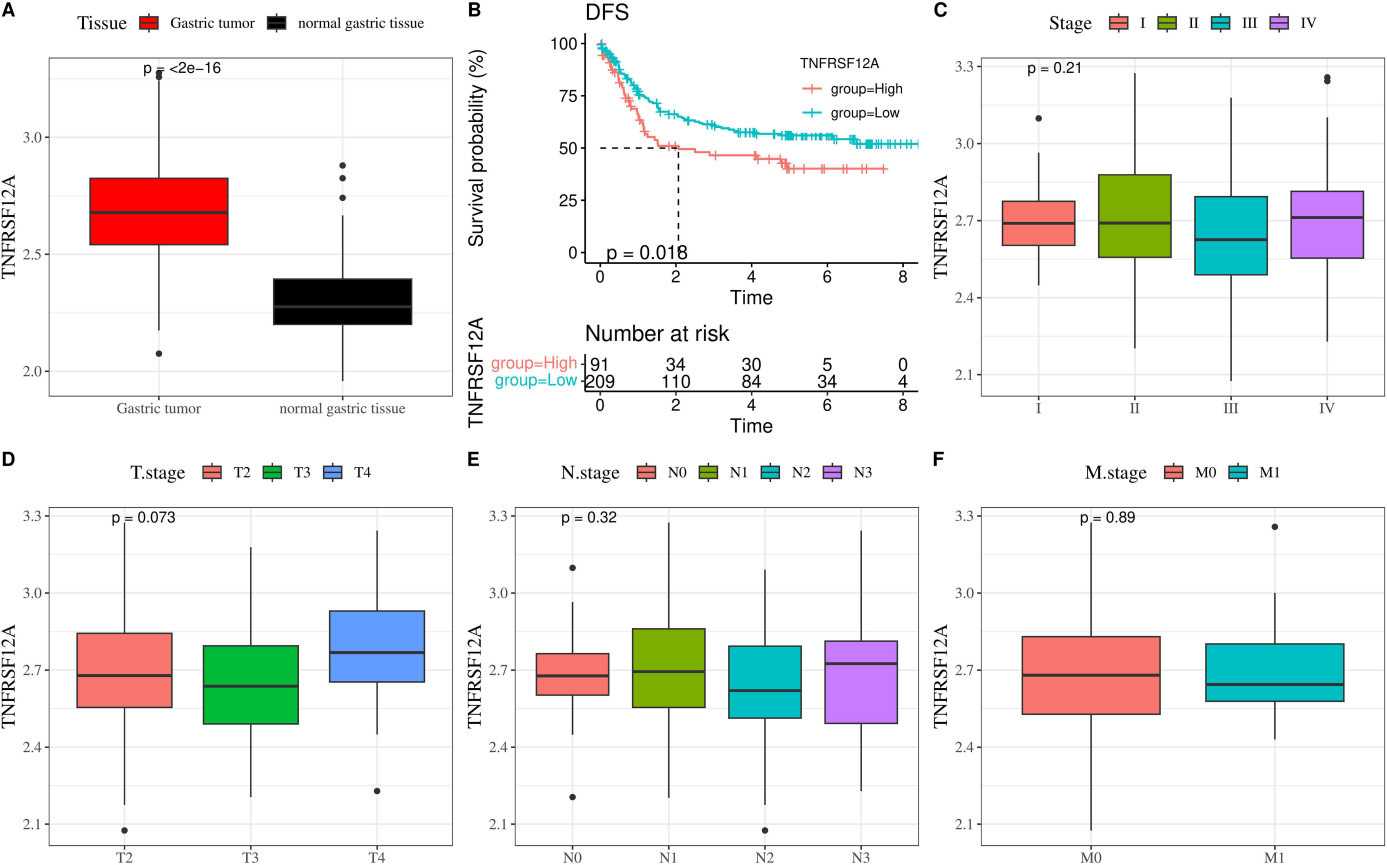
*TNFRSF12A* expression and its relationship with clinical features in GSE66229 dataset. **(A)** Expression level of *TNFRSF12A* in STAD tissue and normal gastric tissue; **(B)** K-M curve of disease-free survival (DFS) for high and low *TNFRSF12A* expression groups; **(C)** Correlation between *TNFRSF12A* expression and Stage; **(D)** Correlation between *TNFRSF12A* expression and T stage; **(E)** Correlation between *TNFRSF12A* expression and N stage; **(F)** Correlation between *TNFRSF12A* expression and M stage.

We further analyzed the relationship of *TNFRSF12A* with prognosis and clinicopathologic features in GSE66229 dataset. The expression of *TNFRSF12A* in STAD tissue was markedly higher than that in normal gastric tissue (**Figure 2A**). Based on K-M survival analysis, high *TNFRSF12A* expression group exhibited a lower disease-free survival (DFS) probability than low *TNFRSF12A* expression group (**Figure 2B**), indicating that STAD patients with a high *TNFRSF12A* expression may have a worse prognosis. Furthermore, there was no significant difference of *TNFRSF12A* expression in different Stage (I-IV), T stages, N stages, and M stages (*p*>0.05) (**Figures 2C–F**). This result implies that the *TNFRSF12A* high-expression group possessed a worse prognosis, but there was no significant difference in *TNFRSF12A* expression in STAD patients with different stages.

### 3.3 Correlation between *TNFRSF12A* expression and biological pathways

**Figure 3:**
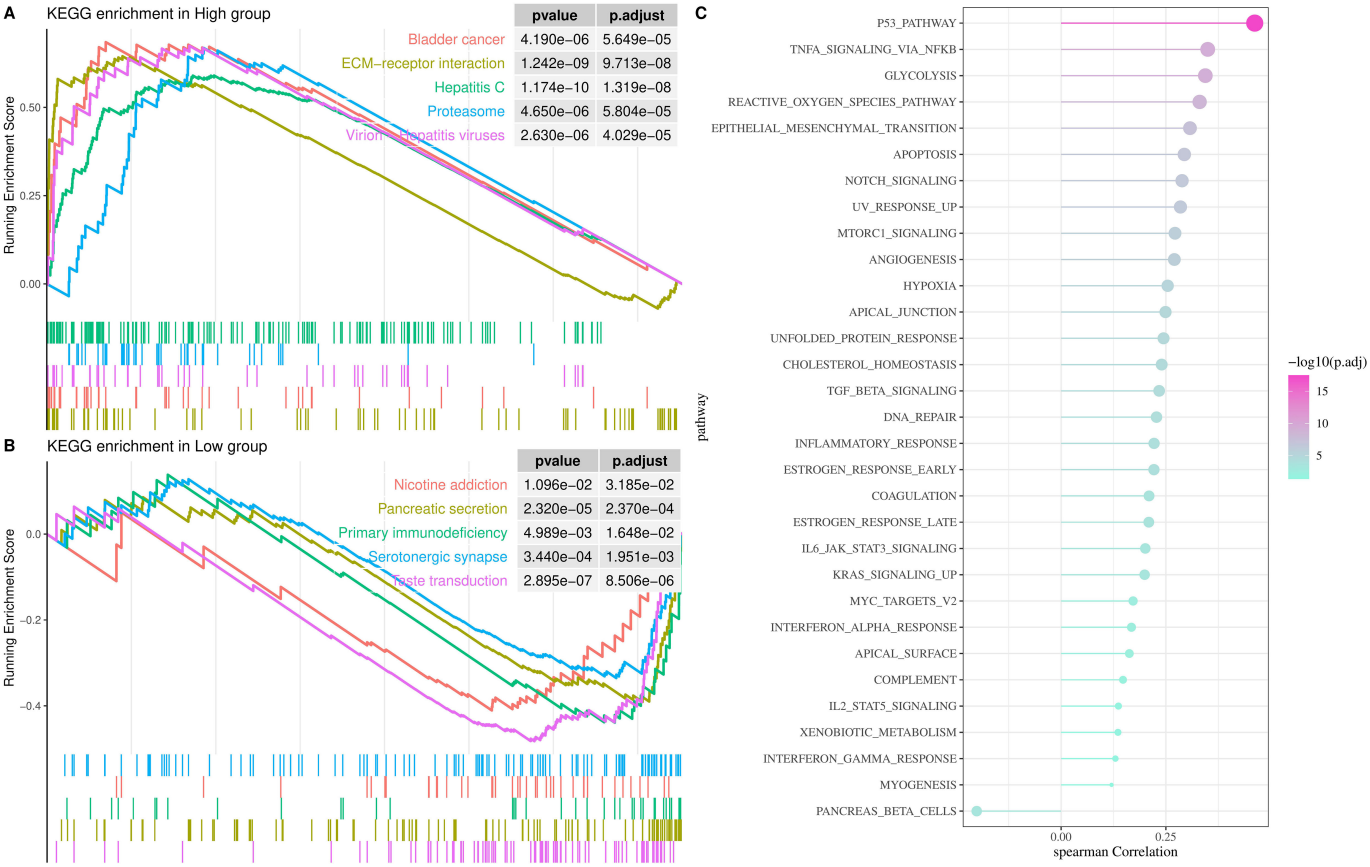
Analysis of biological pathways involved in *TNFRSF12A* in TCGA-STAD cohort. **(A)** KEGG enrichment pathways for high *TNFRSF12A* expression group; **(B)** KEGG enrichment pathways for low *TNFRSF12A* expression group; **(C)** Correlation between *TNFRSF12A* expression and HALLMARK pathways.

Biological pathways involved in *TNFRSF12A* were identified by GSEA, and the samples in TCGA-STAD cohort were split into high and low groups according to the median expression level of *TNFRSF12A*. KEGG enrichment analysis demonstrated that the high expression group mainly participated in the pathways of Bladder cancer, extracellular matrix (ECM)-receptor interaction, Hepatitis C, Proteasome, and Virion-Hepatitis viruses (**Figure 3A**). Whereas, low expression group was principally enriched in the Nicotine addiction, Pancreatic secretion, Primary immunodeficiency, Serotonergic synapse, and Taste transduction pathways (**Figure 3B**). Additionally, the relationship between *TNFRSF12A* expression and HALLMARK pathways was analyzed, suggesting that *TNFRSF12A* exhibited notably positive correlation with many carcinogenic signaling pathways, such as reactive oxygen species (ROS) pathway, TNFA signaling via NFKB, P53 pathway, glycolysis, epithelial-mesenchymal transition (EMT), etc (**Figure 3C**). These results provided new lights on the potential role of *TNFRSF12A* in STAD. This result shows that *TNFRSF12A* is closely associated with oncogenic signaling pathways, suggesting its potential as a biomarker.

### 3.4 Relationship between *TNFRSF12A* expression and immune cell infiltration

**Figure 4:**
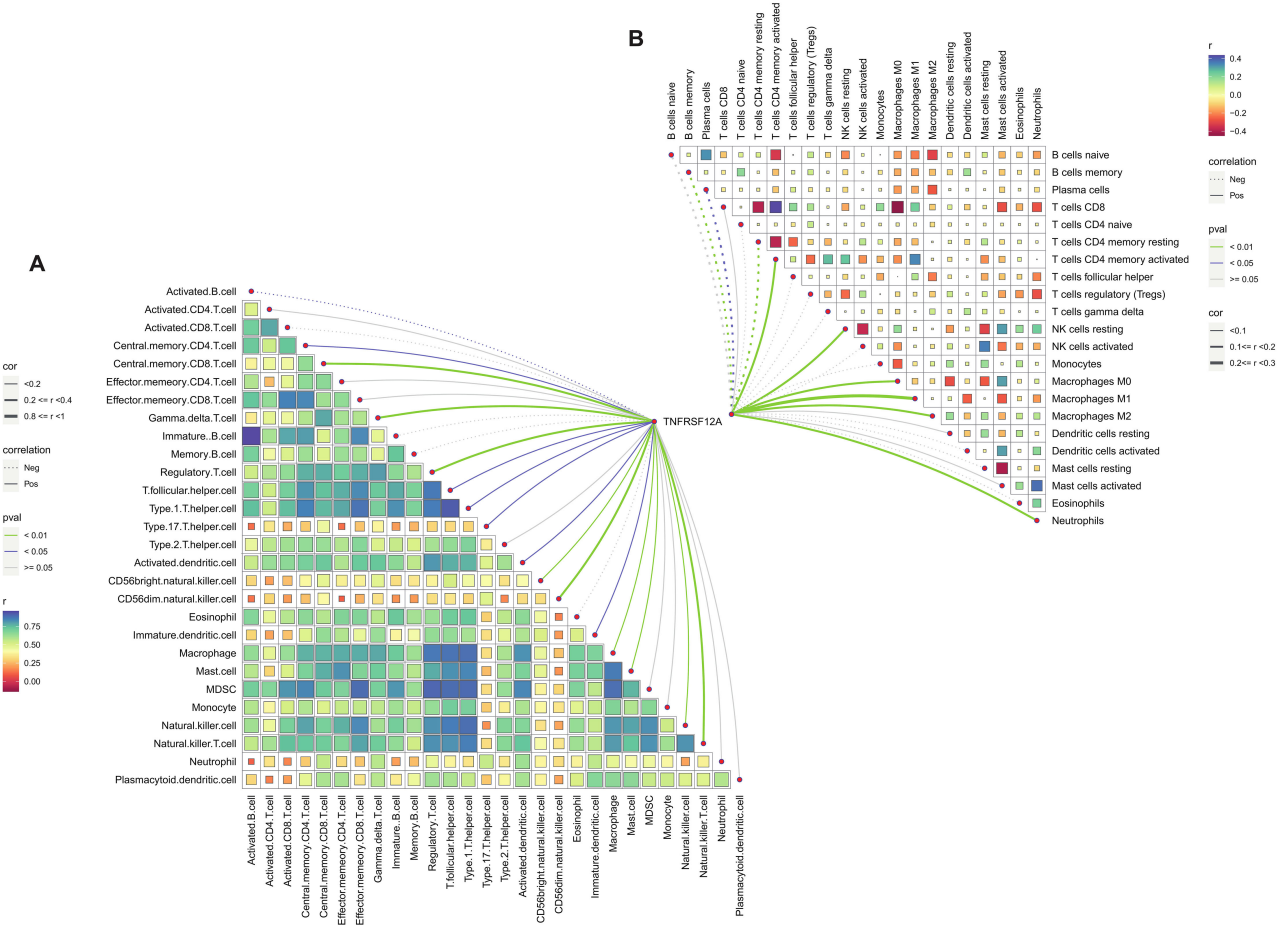
Correlation analysis of *TNFRSF12A* expression and immune cell infiltration. **(A)** Correlation between *TNFRSF12A* and 28 tumor-infiltrating lymphocytes (TILs) assessed by ssGSEA; **(B)** Correlation between *TNFRSF12A* and 22 immune cells evaluated by CIBERSORT.

The immune cell infiltration of *TNFRSF12A* in STAD was evaluated by ssGSEA and CIBERSORT algorithm. It was found that *TNFRSF12A* was significantly positively correlated with the infiltration of numerous immune cells, comprising Regulatory T cell, central memory CD8 T cell, T follicular helper cell, activated Dendritic cell, Natural Killer (NK) cell, Macrophage, Mast cells (MCs), central memory CD4 T cell, NKT cell, Neutrophils, and so on (**Figures 4A, B**). These findings manifested that *TNFRSF12A* may exert an essential role in regulating the TME in STAD.

### 3.5 Prediction of immunotherapy responses between high and low *TNFRSF12A* expression groups

**Figure 5:**
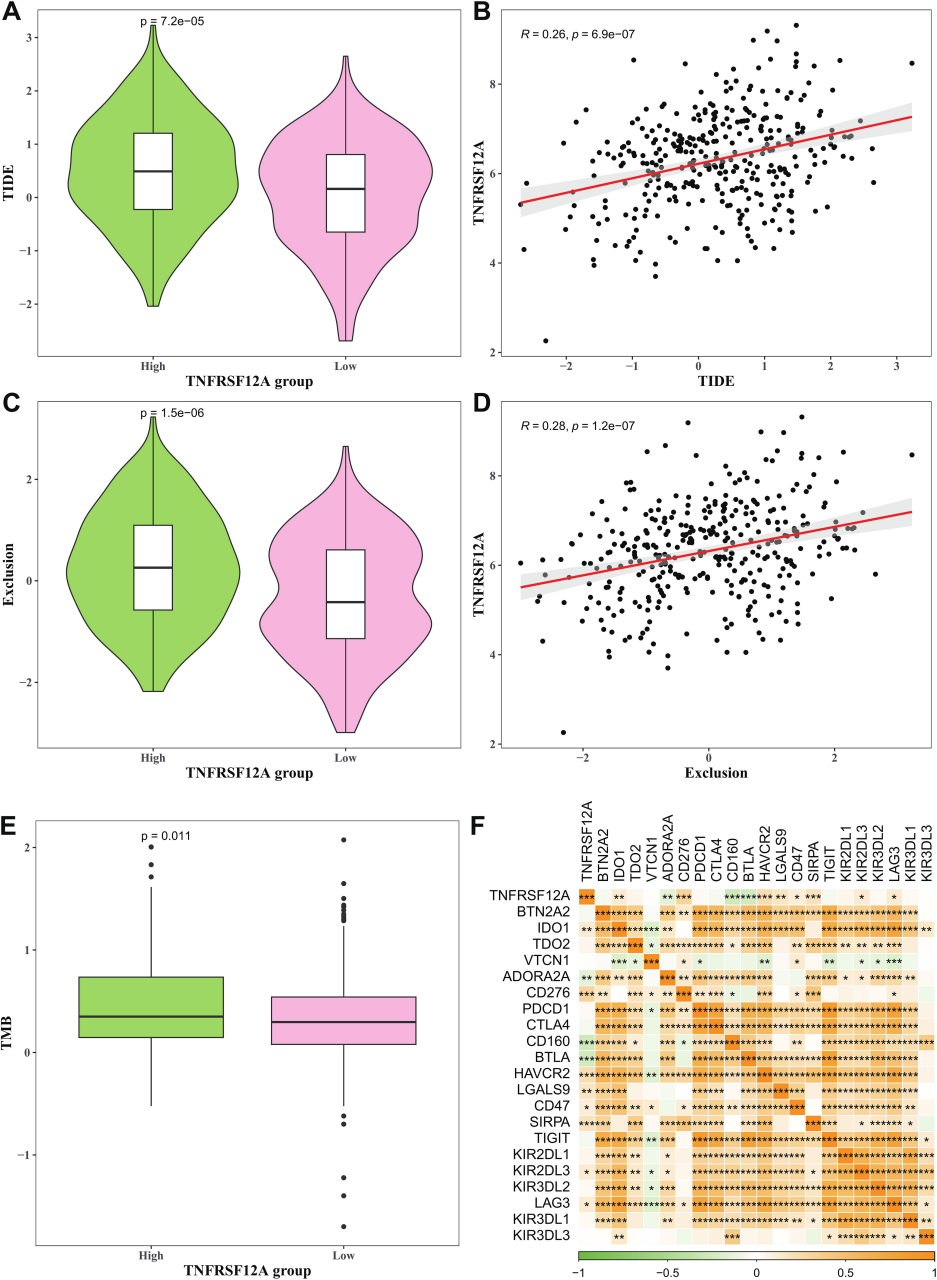
Prediction of immunotherapy response between high and low *TNFRSF12A* expression groups. **(A)** TIDE score in high and low *TNFRSF12A* expression groups; **(B)** Correlation between *TNFRSF12A* and TIDE score; **(C)** Exclusion score in high and low *TNFRSF12A* expression groups; **(D)** Correlation between *TNFRSF12A* and Exclusion score; **(E)** TMB in high and low *TNFRSF12A* expression groups; **(F)** Correlation between *TNFRSF12A* and immune checkpoint genes; *** means *p*<0.001, ** means *p*<0.01, * means *p*<0.05.

TIDE score and Exclusion score of high *TNFRSF12A* expression group were all markedly higher than that of low *TNFRSF12A* expression group (**Figures 5A, C**), and *TNFRSF12A* exhibited positive relationship with TIDE score and Exclusion score (**Figures 5B, D**). This suggested that STAD patients with high *TNFRSF12A* expression might have a higher likelihood of immune escape and less benefit from ICIs therapy. Additionally, high *TNFRSF12A* expression group had a higher TMB compared to low *TNFRSF12A* expression group (**Figure 5E**), which may affect the therapeutic response of STAD patients to ICIs. *TNFRSF12A* was positively correlated with most of the immune checkpoint genes, including *BTN2A2*, *IDO1*, *TDO2*, *ADORA2A*, *PDCD1*, *CTLA4*, *CD160*, *HAVCR2*, *TIGIT*, *KIR2DL3*, etc (**Figure 5F**). These outcomes further demonstrated the important role of *TNFRSF12A* in evaluating the response of STAD patients to ICIs therapy, providing a potential target for clinical application.

### 3.6 Expression of *TNFRSF12A* in the single cell of STAD

**Figure 6:**
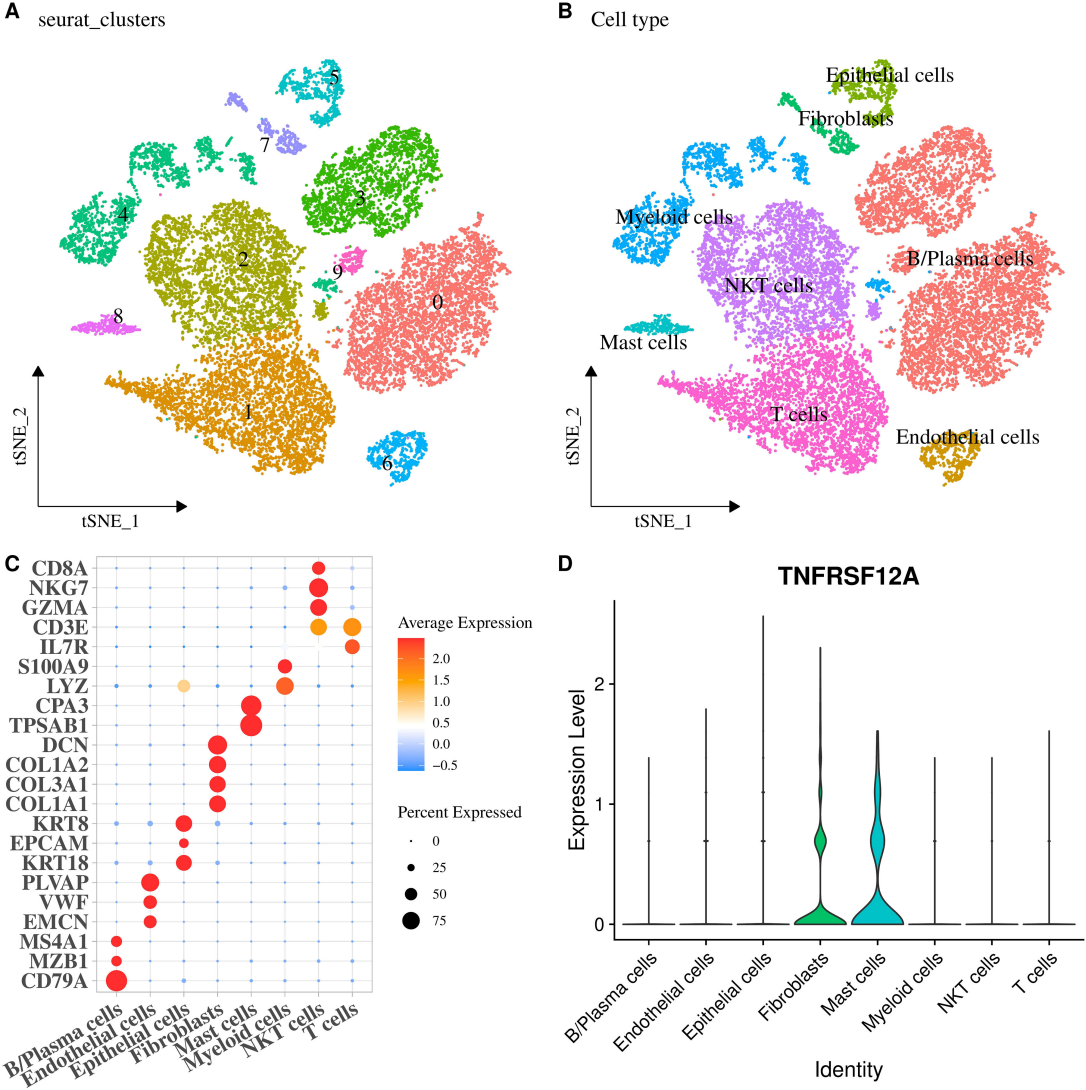
Expression of *TNFRSF12A* in the single cell of STAD. **(A)** The tSNE plot of cell clusters in STAD; **(B)** The tSNE plot of cell types in STAD; **(C)** Bubble diagram of marker genes expression in each cell type; **(D)** Expression levels of *TNFRSF12A* in each cell type.

The scRNA-seq data of STAD in GSE167297 dataset was analyzed, revealing 10 cell clusters (**Figure 6A**). Then, 8 cell types were determined (**Figure 6B**), containing B/Plasma cells (*CD79A*, *MZB1*, *MS4A1*), Endothelial cells (*EMCN*, *VWF*, *PLVAP*), Epithelial cells (*KRT18*, *EPCAM*, *KRT8*), Fibroblasts (*COL1A1*, *COL3A1*, *COL1A2*, *DCN*), Mast cells (*TPSAB1*, *CPA3*), Myeloid cells (*LYZ*, *S100A9*), NKT cells (*CD8A*, *NKG7*, *GZMA*), and T cells (*CD3E*, *IL7R*) (**Figure 6C**). Further, the expression level of *TNFRSF12A* in each cell type was displayed by a violin plot, discovering that *TNFRSF12A* was primarily expressed in Fibroblasts and Mast cells (**Figure 6D**). Hence, we selected the marker genes of Fibroblasts (*DCN*) and Mast cells (*TPSAB1*) for subsequent *in vitro* validation assays.

### 3.7 *TNFRSF12A* silencing notably suppressed the migratory and invasive abilities of STAD cells

**Figure 7:**
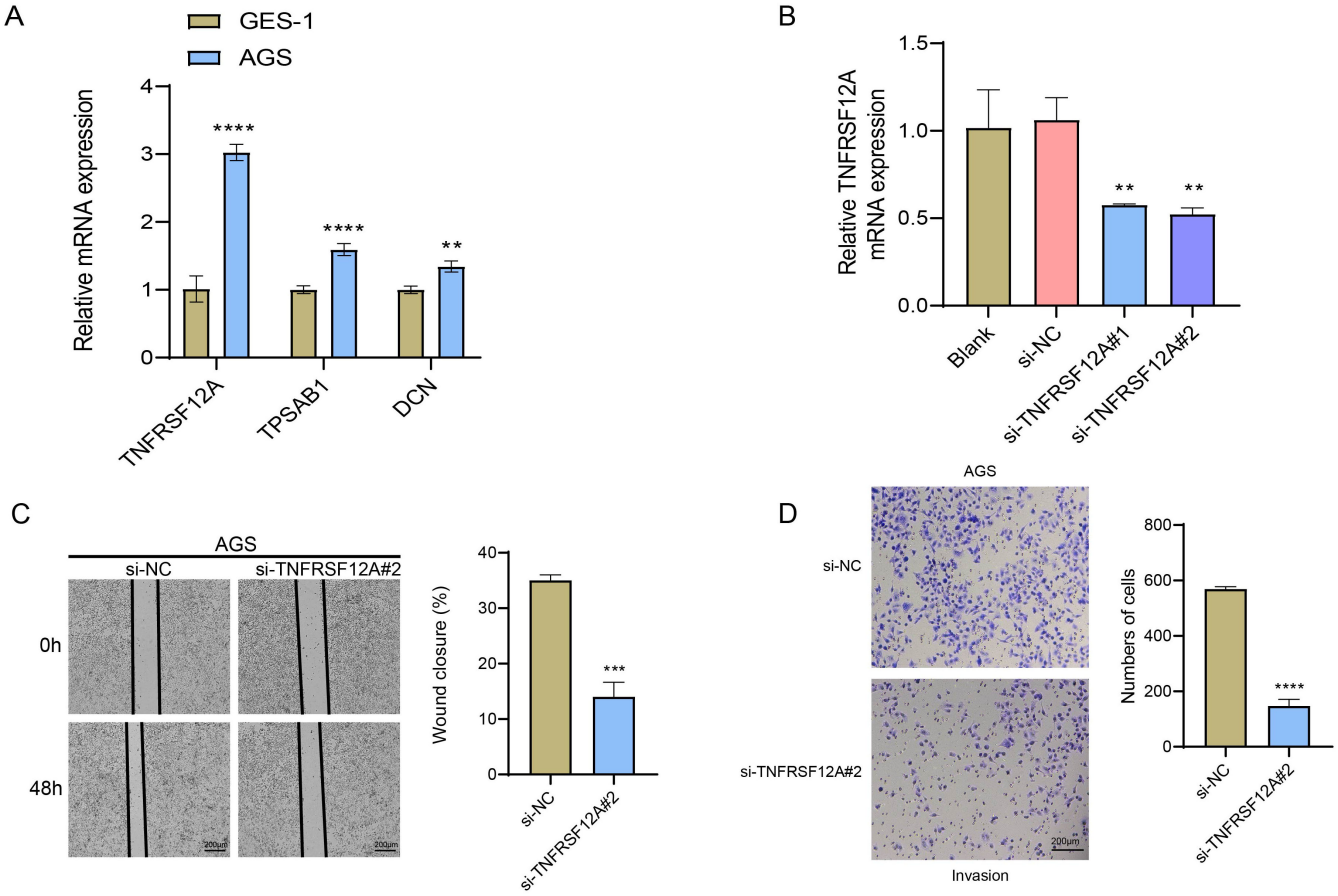
*In vitro* verification utilizing STAD cells. **(A)** Relative mRNA expression levels of *TNFRSF12A*, *TPSAB1*, and *DCN* in human gastric mucosa epithelial cell line GES-1 and STAD cell line AGS detected by RT−qPCR; **(B)** RT-qPCR to verify the success of *TNFRSF12A* transfection; **(C)** Cell migration of *TNFRSF12A*-silenced AGS assessed by Wound healing assay; **(D)** Cell invasion of *TNFRSF12A*-silenced AGS evaluated by Transwell assay. And ** indicates *p*<0.01, *** indicates *p*<0.001, **** indicates *p*<0.0001.

The relative mRNA expressions of *TNFRSF12A*, *TPSAB1*, and *DCN* in human gastric mucosa epithelial cell line GES-1 and STAD cell line AGS were detected through RT−qPCR. It was found that compared with GES-1 cells, *TNFRSF12A*, *TPSAB1*, and *DCN* were all highly expressed in AGS cells (**Figure 7A**). To perform siRNA transfection targeting *TNFRSF12A* while minimizing off-target effects, two targeting sequences were selected. The results of RT−qPCR confirmed the success of the transfection (*p*<0.05) (**Figure 7B**). Subsequently, si-*TNFRSF12A*#2 was chosen for the subsequent experiments. Thereafter, Wound healing assay displayed that the wound closure rate of AGS cells was significantly decreased via *TNFRSF12A* silencing (**Figure 7C**). Moreover, Transwell assay revealed that the number of invaded AGS cells was markedly declined via *TNFRSF12A* silencing (**Figure 7D**). These data supported that *TNFRSF12A* functioned crucially in STAD cell migration and invasion, which may be a promising target for controlling STAD progression.

## 4 Discussion

*TNFRSF12A* is expressed in various human tissues, containing liver, heart, lung, and skeletal muscle (37). Numerous evidences have manifested that the dysregulation of *TNFRSF12A* plays an important part in the triggering and development of malignant tumors (38). *TNFRSF12A* is also found to be overexpressed in various carcinomas, which usually indicates a poor prognosis (39). In this study, we verified the high expression of *TNFRSF12A* in STAD by bioinformatics as well as *in vitro* experiments and found that it may affect STAD immunotherapy by influencing immune infiltration, a finding that provides a new therapeutic target for personalized treatment of STAD.

The biological pathways involved in *TNFRSF12A* were analyzed by GSEA in this study, suggesting that *TNFRSF12A* was positively related to many carcinogenic signaling pathways, such as P53 pathway, glycolysis, TNFA signaling via NFKB, ROS pathway, EMT, and so on. P53 as a tumor suppressor is mutated in around 50% of STAD, and these mutations are more frequent in intestinal tumors than diffuse tumors (40). The gene set “TNFA signaling via NFKB” contains 182 genes, such as TNF-α, NFKB, and the other inflammatory cytokines (41). TNF-α and NFKB are relevant to several important biological processes, including inflammatory response, immune regulation, tumorigenesis, and tumor cell apoptosis (42). In addition, glycolysis pathway as the main source of energy acquisition for cancer tissues significantly impacts the tumor growth, invasiveness, chemotherapy resistance, TME, and immune escape (43). Elevated glycolysis belongs to a part of the “Warburg effect”, enabling STAD cells to produce lactic acid, which provides energy for cell biosynthesis and cell division (44). Oxidative stress represents a disorder of antioxidant defense system implicated in the production of excessive ROS in tumor cells, which is connected with the angiogenesis, DNA damage, and tumor metastasis (45). Besides, EMT is a crucial process in the development of epithelial malignancies, including STAD, promoting the migration and invasion of cancer cells (46, 47). Hence, these carcinogenic signaling pathways may exert a crucial role in the triggering of STAD and its development, and these data can offer new clues to the potential role of *TNFRSF12A* in STAD.

Data from single-cell analyses show that *TNFRSF12A* was expressed predominantly in fibroblasts and mast cells. Santi et al. used the expression levels of α-SMA and *TNFRSF12A* to differentiate between cancer associated fibroblast (CAF) subpopulations (48). TNFRSF12A^+^ CAF plays a key role in the immunosuppression of intestinal-type gastric adenocarcinoma (IGAC) (a type of STAD) acts as a key mediator in immunosuppression and has potential as an immunomodulator (48). Furthermore, *TNFRSF12A* was found to act as a C2 ALOX5^+^ MCs and tumor cell key receptor in the communication pathway that plays a role in cervical cancer progression (49). These evidences imply that *TNFRSF12A* in CAF and MC may influence the development of STAD.

An increasing number of researches have proved that immune cell infiltration in TME is closely relevant to patient prognosis and conducive to the prediction of immunotherapy response in STAD (50). In this present study, we found that *TNFRSF12A* was notably positively related to the infiltration of most immune cells in STAD, for instance central memory CD4 T cell, Macrophage, Regulatory T cell, T follicular helper cell, central NK cell, memory CD8 T cell, NKT cell, activated Dendritic cell, Mast cell, Neutrophils. Macrophage serves as an important immunosuppressive cell and hampers the activation of NK cells and CD8 T cells (51). Mast cell can promote tumor proliferation and invasion directly, or indirectly via regulating TME (52). A study of tumor-bearing mice has indicated that Neutrophils can facilitate the tumorigenesis and aggressiveness of GC cells by mediating EMT (53). Thus, *TNFRSF12A* may play a pivotal role in regulating the TME in STAD. Furthermore, TIDE score and TMB are widely utilized as predictive indicators for STAD patients during ICIs therapy, contributing to the clinical decision-making (54). In this study, we found that the TIDE score, Exclusion score, and TMB level of high *TNFRSF12A* expression group were all markedly higher than that of low *TNFRSF12A* expression group, demonstrating that STAD patients with high *TNFRSF12A* expression might have stronger immune escape and poorer response to immunotherapy (55). In addition, this study demonstrated that *TNFRSF12A* was positively correlated with most of the immune checkpoint genes, and previous studies have also found that silencing of *TNFRSF12A* can inhibit GC cell viability, increase T cell proliferation, and affect the NF-kB pathway (21). These findings imply that *TNFRSF12A* is highly likely to have an important impact on the body’s immune response by regulating the proliferation process of T cells, which in turn suggests its potential role in the field of tumor immunotherapy. Taken together, these outcomes further emphasized the important role of *TNFRSF12A* in STAD, providing a promising therapeutic target for clinical application.

## 5 Conclusion

To conclude, the present work discovered that *TNFRSF12A* was high-expressed in STAD, which was linked to the low survival rate and poor prognosis. *TNFRSF12A* showed the positive correlation with many carcinogenic signaling pathways and immune cells infiltration. STAD patients with high *TNFRSF12A* expression may have stronger immune escape and poorer immunotherapy response. These outcomes could provide a new insight on the role of *TNFRSF12A* in STAD and supply a potential therapeutic target for STAD.

## Data Availability

The datasets presented in this study can be found in online repositories. The names of the repository/repositories and accession number(s) can be found in the article/supplementary material.

## Glossary

COAD: colon adenocarcinoma
DFS: disease-free survival
ECM: extracellular matrix
EMT: epithelial-mesenchymal transition
FBS: fetal bovine serum
FPKM: fragments per kilobase of transcript per million fragments mapped
GBM: glioblastoma
GC: gastric cancer
GEO: Gene Expression Omnibus
GEPIA: Gene Expression Profiling Interactive Analysis
GSEA: gene set enrichment analysis
ICIs: immune checkpoint inhibitors
KEGG: Kyoto Encyclopedia of Genes and Genomes
K-M: Kaplan-Meier
LIHC: liver hepatocellular carcinoma
LUAD: lung adenocarcinoma
MSigDB: Molecular Signatures Database
NES: normalized enrichment score
NFKB: nuclear factor kappa B
NK: Natural Killer
OS: overall survival
PFS: progression-free survival
RNA-seq: RNA sequencing
ANOVA: analysis of variance
ROS: reactive oxygen species
RT−qPCR: real time quantitative PCR
scRNA-seq: single-cell RNA sequencing
si: small interfering
ssGSEA: single sample gene set enrichment analysis
STAD: stomach adenocarcinoma
TCGA: the Cancer Gene Atlas
TIDE: Tumor Immune Dysfunction and Exclusion
TILs: tumor-infiltrating lymphocytes
TMB: tumor mutation burden
TME: tumor microenvironment
TNF: tumor necrosis factor
TPM: transcripts per million
tSNE: t-Distributed Stochastic Neighbor Embedding
CAF: cancer associated fibroblast
MC: mast cell
IGAC: intestinal-type gastric adenocarcinoma
STR: short tandem repeat

## References

[1] Su X Zhang J Luo X Yang W Liu Y Liu Y. LncRNA LINC01116 promotes cancer cell proliferation, migration and invasion in gastric cancer by positively interacting with lncRNA CASC11. OncoTargets Ther. (2019) 12:8117–23. doi: 10.2147/OTT.S208133 PMC6781852 31632064

[2] Fatima S Song Y Zhang Z Fu Y Zhao R Malik K. Exploring the pharmacological mechanisms of P-hydroxylcinnamaldehyde for treating gastric cancer: A pharmacological perspective with experimental confirmation. Curr Mol Pharmacol. (2024) 17:e18761429322420. doi: 10.2174/0118761429322420241112051105 39660529

[3] Jaroenlapnopparat A Bhatia K Coban S. Inflammation and gastric cancer. Dis (Basel Switzerland). (2022) 10:35. doi: 10.3390/diseases10030035 PMC9326573 35892729

[4] Chandarana CV Mithani NT Singh DV Kikani UB. Vibrational spectrophotometry: A comprehensive review on the diagnosis of gastric and liver cancer. Curr Pharm Anal. (2024) 20:453–65. doi: 10.2174/0115734129322567240821052326

[5] Song J Xu X He S Wang N Bai Y Chen Z. Myristicin suppresses gastric cancer growth via targeting the EGFR/ERK signaling pathway. Curr Mol Pharmacol. (2023) 16(7):712–24. doi: 10.2174/1874467216666230103104600 36597605

[6] Wang H Luo Y Chu Z Ni T Ou S Dai X. Poria acid, triterpenoids extracted from Poria cocos, inhibits the invasion and metastasis of gastric cancer cells. Molecules (Basel Switzerland). (2022) 27:3629. doi: 10.3390/molecules27113629 35684565 PMC9182142

[7] Luo WZ Li X Wu XX Shang YW Meng DH Chen YL. MAGED4B is a poor prognostic marker of stomach adenocarcinoma and a potential therapeutic target for stomach adenocarcinoma tumorigenesis. Int J Gen Med. (2023) 16:1681–93. doi: 10.2147/IJGM.S401507 PMC10171223 37181643

[8] Zhou X Nie H Wang C Yu X Yang X He X. Prognostic value and therapeutic potential of NEK family in stomach adenocarcinoma. J Cancer. (2024) 15:3154–72. doi: 10.7150/jca.90197 PMC11064251 38706902

[9] Yu P Chen P Wu M Ding G Bao H Du Y. Multi-dimensional cell-free DNA-based liquid biopsy for sensitive early detection of gastric cancer. Genome Med. (2024) 16:79. doi: 10.1186/s13073-024-01352-1 38849905 PMC11157707

[10] Jin C Lu X Yang M Hou S. Integrative analysis indicates the potential values of ANKRD53 in stomach adenocarcinoma. Discover Oncol. (2024) 15:188. doi: 10.1007/s12672-024-01054-5 PMC11130106 38801557

[11] Chen H Zheng Z Yang C Tan T Jiang Y Xue W. Machine learning based intratumor heterogeneity signature for predicting prognosis and immunotherapy benefit in stomach adenocarcinoma. Sci Rep. (2024) 14:23328. doi: 10.1038/s41598-024-74907-2 39375438 PMC11458769

[12] Yang J Jin F Li H Shen Y Shi W Wang L. Identification of mitochondrial respiratory chain signature for predicting prognosis and immunotherapy response in stomach adenocarcinoma. Cancer Cell Int. (2023) 23:69. doi: 10.1186/s12935-023-02913-x 37062830 PMC10105960

[13] Ren N Liang B Li Y. Identification of prognosis-related genes in the tumor microenvironment of stomach adenocarcinoma by TCGA and GEO datasets. Bioscience Rep. (2020) 40:BSR20200980. doi: 10.1042/BSR20200980 PMC7560520 33015704

[14] Salleh EA Lee YY Zakaria AD Jalil NAC Musa M. Cancer-associated fibroblasts of colorectal cancer: Translational prospects in liquid biopsy and targeted therapy. Biocell. (2023) 47:2233–44. doi: 10.32604/biocell.2023.030541

[15] Qiu H Zhang X Yu H Gao R Shi J Shen T. Identification of potential targets of triptolide in regulating the tumor microenvironment of stomach adenocarcinoma patients using bioinformatics. Bioengineered. (2021) 12:4304–19. doi: 10.1080/21655979.2021.1945522 PMC8806726 34348580

[16] Xiong X Hu T Yin Z Zhang Y Chen F Lei P. Research advances in the study of sleep disorders, circadian rhythm disturbances and Alzheimer’s disease. Front Aging Neurosci. (2022) 14. doi: 10.3389/fnagi.2022.944283 PMC9428322 36062143

[17] Liao M Liao J Qu J Shi P Cheng Y Pan Q. Hepatic TNFRSF12A promotes bile acid-induced hepatocyte pyroptosis through NFκB/Caspase-1/GSDMD signaling in cholestasis. Cell Death disease. (2023) 9:26. doi: 10.1038/s41420-023-01326-z PMC9871041 36690641

[18] Wang C Zhao Y Chen Y Shi Y Yang Z Wu W. The oncogenic role of TNFRSF12A in colorectal cancer and pan-cancer bioinformatics analysis. Cancer Res Treat. (2024) 57:212–28. doi: 10.4143/crt.2024.408 PMC11729321 39118523

[19] Zhang Y Yang X Zhu XL Wang ZZ Bai H Zhang JJ. A novel immune-related prognostic biomarker and target associated with Malignant progression of glioma. Front Oncol. (2021) 11:643159. doi: 10.3389/fonc.2021.643159 33937046 PMC8085360

[20] Yang J Min KW Kim DH Son BK Moon KM Wi YC. High TNFRSF12A level associated with MMP-9 overexpression is linked to poor prognosis in breast cancer: Gene set enrichment analysis and validation in large-scale cohorts. PLoS One. (2018) 13:e0202113. doi: 10.1371/journal.pone.0202113 30142200 PMC6108472

[21] Xia L Jiang L Chen Y Zhang G Chen L. ThPOK transcriptionally inactivates TNFRSF12A to increase the proliferation of T cells with the involvement of the NF-kB pathway. Cytokine. (2021) 148:155658. doi: 10.1016/j.cyto.2021.155658 34353698

[22] Song Z Yu J Wang M Shen W Wang C Lu T. CHDTEPDB: transcriptome expression profile database and interactive analysis platform for congenital heart disease. Congenit Heart Dis. (2023) 18:693–701. doi: 10.32604/chd.2024.048081

[23] Zhang D Wang M Peng L Yang X Li K Yin H. Identification and validation of three PDAC subtypes and individualized GSVA immune pathway-related prognostic risk score formula in pancreatic ductal adenocarcinoma patients. J Oncol. (2021) 2021:4986227. doi: 10.1155/2021/4986227 34987579 PMC8723862

[24] Yuan L Hu C Yu P Bao Z Xia Y Zhang B. High HDAC5 expression correlates with a poor prognosis and the tumor immune microenvironment in gastric cancer. Ann Trans Med. (2022) 10:990. doi: 10.21037/atm-22-4325 PMC9577731 36267769

[25] Charoentong P Finotello F Angelova M Mayer C Efremova M Rieder D. Pan-cancer immunogenomic analyses reveal genotype-immunophenotype relationships and predictors of response to checkpoint blockade. Cell Rep. (2017) 18:248–62. doi: 10.1016/j.celrep.2016.12.019 28052254

[26] Yuan G Jia J Wang Z Xia Y Pan Y Zhang L. Weighted gene co-expression network analysis of immune infiltration in nonalcoholic fatty liver disease. Endocrine Metab Immune Disord - Drug Targets. (2023) 23:1173–85. doi: 10.2174/1871530323666221208105720 36503447

[27] Li X Lei J Shi Y Peng Z Gong M Shu X. Developing a RiskScore Model based on Angiogenesis-related lncRNAs for Colon Adenocarcinoma Prognostic Prediction. Curr Med Chem. (2024) 31:2449–66. doi: 10.2174/0109298673277243231108071620 37961859

[28] Deng Y Lin Y Zhou B Jing Q Zhang W. Identification of necroptosis-related subtypes and characterization of tumor microenvironment infiltration in non-small cell lung cancer. Curr Cancer Drug Targets. (2024) 24:80–93. doi: 10.2174/1568009623666230414140609 37469156

[29] Zulibiya A Wen J Yu H Chen X Xu L Ma X. Single-cell RNA sequencing reveals potential for endothelial-to-mesenchymal transition in tetralogy of fallot. Congenit Heart Dis. (2023) 18:611–25. doi: 10.32604/chd.2023.047689

[30] Chai R Zhao Y Su Z Liang W. Integrative analysis reveals a four-gene signature for predicting survival and immunotherapy response in colon cancer patients using bulk and single-cell RNA-seq data. Front Oncol. (2023) 13:1277084. doi: 10.3389/fonc.2023.1277084 38023180 PMC10644708

[31] Hu C Li T Xu Y Zhang X Li F Bai J. CellMarker 2.0: an updated database of manually curated cell markers in human/mouse and web tools based on scRNA-seq data. Nucleic Acids Res. (2023) 51:D870–d6. doi: 10.1093/nar/gkac947 PMC9825416 36300619

[32] Hou B Zhao LI Ianhao Zhao T Yang M Zhu W Chen X. Chrysophanol inhibits the progression of gastric cancer by activating nod-like receptor protein-3. Biocell. (2023) 47:175–86. doi: 10.32604/biocell.2022.021359

[33] Ou L Liu H Peng C Zou Y Jia J Li H. Helicobacter pylori infection facilitates cell migration and potentially impact clinical outcomes in gastric cancer. Heliyon. (2024) 10:e37046. doi: 10.1016/j.heliyon.2024.e37046 39286209 PMC11402937

[34] Song J Fu Q Liu G Zhang C Wang Y Tao S. TULP3 silencing suppresses cell proliferation, migration and invasion in gastric cancer via the PTEN/Akt/Snail pathway. Cancer Treat Res Commun. (2022) 31:100551. doi: 10.1016/j.ctarc.2022.100551 35344762

[35] Wang W Baral S Liu B Sun Q Wang L Ren J. FANCA facilitates G1/S cell cycle advancement, proliferation, migration and invasion in gastric cancer. Acta Biochim Biophys Sinica. (2024) 56:973–85. doi: 10.3724/abbs.2024045 PMC11322876 38682160

[36] Li S Chen S Wang B Zhang L Su Y Zhang X. A robust 6-lncRNA prognostic signature for predicting the prognosis of patients with colorectal cancer metastasis. Front Med. (2020) 7:56. doi: 10.3389/fmed.2020.00056 PMC7068734 32211413

[37] Ruiz BI Lowman XH Yang Y Fan Q Wang T Wu H. Alpha-ketoglutarate regulates Tnfrsf12a/Fn14 expression via histone modification and prevents cancer-induced cachexia. Genes. (2023) 14:1818. doi: 10.3390/genes14091818 37761958 PMC10531467

[38] Liu JY Jiang L He T Liu JJ Fan JY Xu XH. NETO2 promotes invasion and metastasis of gastric cancer cells via activation of PI3K/Akt/NF-κB/Snail axis and predicts outcome of the patients. Cell Death disease. (2019) 10:162. doi: 10.1038/s41419-019-1388-5 30770791 PMC6377647

[39] Wang Y Zhang S Xie X Chen Z Wu L Yu Z. Association of TNFRSF12A methylation with prognosis in hepatocellular carcinoma with history of alcohol consumption. Front Genet. (2019) 10:1299. doi: 10.3389/fgene.2019.01299 31998364 PMC6964049

[40] Till JE Yoon C Kim BJ Roby K Addai P Jonokuchi E. Oncogenic KRAS and p53 loss drive gastric tumorigenesis in mice that can be attenuated by E-cadherin expression. Cancer Res. (2017) 77:5349–59. doi: 10.1158/0008-5472.CAN-17-0061 PMC5626624 28760854

[41] Subramanian A Tamayo P Mootha VK Mukherjee S Ebert BL Gillette MA. Gene set enrichment analysis: a knowledge-based approach for interpreting genome-wide expression profiles. Proc Natl Acad Sci U S A. (2005) 102:15545–50. doi: 10.1073/pnas.0506580102 PMC1239896 16199517

[42] Xue J Chen L Cheng H Song X Shi Y Li L. The identification and validation of hub genes associated with acute myocardial infarction using weighted gene co-expression network analysis. J Cardiovasc Dev Dis. (2022) 9:30. doi: 10.3390/jcdd9010030 35050240 PMC8778825

[43] Liao Z Xie Z. Construction of a disulfidptosis-related glycolysis gene risk model to predict the prognosis and immune infiltration analysis of gastric adenocarcinoma. Clin Trans Oncol. (2024) 26:2309–22. doi: 10.1007/s12094-024-03457-w 38587603

[44] Zhong Z Ye Z He G Zhang W Wang J Huang S. Low expression of A-kinase anchor protein 5 predicts poor prognosis in non-mucin producing stomach adenocarcinoma based on TCGA data. Ann Trans Med. (2020) 8:115. doi: 10.21037/atm.2019.12.98 PMC7049022 32175408

[45] Li G Ping M Zhang W Wang Y Zhang Z Su Z. Establishment of the molecular subtypes and a risk model for stomach adenocarcinoma based on genes related to reactive oxygen species. Heliyon. (2024) 10:e27079. doi: 10.1016/j.heliyon.2024.e27079 38463816 PMC10923688

[46] Chen K Xu J Tong YL Yan JF Pan Y Wang WJ. Rab31 promotes metastasis and cisplatin resistance in stomach adenocarcinoma through Twist1-mediated EMT. Cell Death disease. (2023) 14:115. doi: 10.1038/s41419-023-05596-4 36781842 PMC9925739

[47] Xie X Zhou Z Song Y Zhang X Dang C Zhang H. Mist1 inhibits epithelial-mesenchymal transition in gastric adenocarcinoma via downregulating the Wnt/β-catenin pathway. J Cancer. (2021) 12:4574–84. doi: 10.7150/jca.59138 PMC8210560 34149921

[48] Wang Q Chen J Wang Y Li X Ping X Shen J. The profiles of immunosuppressive microenvironment in the Lauren intestinal-type gastric adenocarcinoma. Cancer Immunol Immunother. (2025) 74:82. doi: 10.1007/s00262-024-03938-5 39891785 PMC11787096

[49] Zhao F Hong J Zhou G Huang T Lin Z Zhang Y. Elucidating the role of tumor-associated ALOX5+ mast cells with transformative function in cervical cancer progression via single-cell RNA sequencing. Front Immunol. (2024) 15:1434450. doi: 10.3389/fimmu.2024.1434450 39224598 PMC11366577

[50] Luo N Fu M Zhang Y Li X Zhu W Yang F. Prognostic role of M6A-associated immune genes and cluster-related tumor microenvironment analysis: A multi-omics practice in stomach adenocarcinoma. Front Cell Dev Biol. (2022) 10:935135. doi: 10.3389/fcell.2022.935135 35859893 PMC9291731

[51] Xiang Z Cha G Wang Y Gao J Jia J. Characterizing the crosstalk of NCAPG with tumor microenvironment and tumor stemness in stomach adenocarcinoma. Stem Cells Int. (2022) 2022:1888358. doi: 10.1155/2022/1888358 36238529 PMC9551677

[52] Wang M Li Z Peng Y Fang J Fang T Wu J. Identification of immune cells and mRNA associated with prognosis of gastric cancer. BMC cancer. (2020) 20:206. doi: 10.1186/s12885-020-6702-1 32164594 PMC7068972

[53] Wang Y Li X Zhang T Li F Shen Y He Y. Neutrophils promote tumor invasion via FAM3C-mediated epithelial-to-mesenchymal transition in gastric cancer. Int J Biol Sci. (2023) 19:1352–68. doi: 10.7150/ijbs.79022 PMC10086748 37056931

[54] Meijing Z Tianhang L Biao Y. N6-methyladenosine modification patterns and tumor microenvironment immune characteristics associated with clinical prognosis analysis in stomach adenocarcinoma. Front Cell Dev Biol. (2022) 10:913307. doi: 10.3389/fcell.2022.913307 35813200 PMC9261346

[55] Yan P Cheng M Wang L Zhao W. A ferroptosis-related gene in Helicobacter pylori infection, SOCS1, serves as a potential prognostic biomarker and corresponds with tumor immune infiltration in stomach adenocarcinoma: In silico approach. Int immunopharmacol. (2023) 119:110263. doi: 10.1016/j.intimp.2023.110263 37156031

